# Antenna S-parameter optimization based on golden sine mechanism based honey badger algorithm with tent chaos

**DOI:** 10.1016/j.heliyon.2023.e21596

**Published:** 2023-11-04

**Authors:** Oluwatayomi Rereloluwa Adegboye, Afi Kekeli Feda, Meshack Magaji Ishaya, Ephraim Bonah Agyekum, Ki-Chai Kim, Wulfran Fendzi Mbasso, Salah Kamel

**Affiliations:** a Management Information Systems, University of Mediterranean Karpasia, Nicosia, Mersin, 10, Turkey; b Management Information System Department, European University of Lefke, Mersin, 10, Turkey; c Electrical and Electronics Engineering Department, Cyprus International University, Mersin, 10, Turkey; d Department of Nuclear and Renewable Energy, Ural Federal University Named After the First President of Russia Boris, 19 Mira Street, Yeltsin, Ekaterinburg, 620002, Russia; e Department of Electrical Engineering, Yeungnam University, Gyeongsan, 38541, South Korea; f Laboratory of Technology and Applied Sciences, University Institute of Technology, University of Douala, PO Box: 8698, Douala, Cameroon; g Electrical Engineering Department, Faculty of Engineering, Aswan University, 81542, Aswan, Egypt

## Abstract

This work proposed a new method to optimize the antenna S-parameter using a Golden Sine mechanism-based Honey Badger Algorithm that employs Tent chaos (GST-HBA). The Honey Badger Algorithm (HBA) is a promising optimization method that similar to other metaheuristic algorithms, is prone to premature convergence and lacks diversity in the population. The Honey Badger Algorithm is inspired by the behavior of honey badgers who use their sense of smell and honeyguide birds to move toward the honeycomb. Our proposed approach aims to improve the performance of HBA and enhance the accuracy of the optimization process for antenna S-parameter optimization. The approach we propose in this study leverages the strengths of both tent chaos and the golden sine mechanism to achieve fast convergence, population diversity, and a good tradeoff between exploitation and exploration. We begin by testing our approach on 20 standard benchmark functions, and then we apply it to a test suite of 8 S-parameter functions. We perform tests comparing the outcomes to those of other optimization algorithms, the result shows that the suggested algorithm is superior.

## 1 Introduction

In the last couple of decades, numerous metaheuristic algorithms have been introduced to enhance various systems’ functionality and lower the cost of computation. Metaheuristic optimization algorithms have received wide attention largely as a result of their simplicity and flexibility. The following are a few of the most popular metaheuristic algorithms: Simulated Annealing (SA) [1], Bacterial Foraging Optimization algorithm (BFO) [2], Particle Swarm Optimization (PSO) [3], Moth Flame Optimization (MFO) [4], Chimp Optimization Algorithm (ChOA) [5], Whale Optimization Algorithm (WOA) [6], Artificial Bee Colony (ABC) [7], Ant Colony Optimization (ACO) [8], Elephant Herding Optimization (EHO) [9], Harris Hawks Optimization (HHO) [10], Differential Evolution (DE) [11], Crow Search Algorithm (CSA) [12], Grasshopper Optimization Algorithm (GOA) [13], Honey Badger Algorithm (HBA) [14] and Artificial Electric Field Algorithm (AEFA) [15]. There is currently a large use of metaheuristic optimization algorithms in numerous aspects of real life as technology continues to advance. They are used for pathfinding [16], neural network optimization [17], water distribution systems [18], trajectory optimization [19], power flow problem [20], intrusion detection [21], medical feature selection [22], heat and power economic dispatch problem [23], parameter estimation of photovoltaic cells [24], predictive modeling [25], reliability–redundancy allocation problem [26], bridge maintenance [27], image segmentation [28], the welding shop scheduling problem [29], tuning of fuzzy control systems [30], home health care supply chain [31] and antenna parameter optimization [32].

The optimization of antenna design parameters has recently attracted a lot of attention. The topological complexity of modern antenna constructions has progressively risen over time to satisfy ever-stricter requirements. In the sphere of communication, fine-tuning the geometric and material features of antenna design structures to meet these requirements has become a standard [33]. The most popular methods for fine-tuning antenna parameters involve trial-and-error procedures based on scanning a number of design parameters using experience-based methods. These methods require a significant amount of time, and their success is not guaranteed, henceforth the need for optimization-based design automation. The main methods used to improve antenna performance through optimization are local and global numerical approaches. Despite the fact that numerical optimization is preferable to parameter scanning using experience-based methods, some challenges remain [34]. In order to obtain effective and relevant results, local optimization approaches generally require an adequate starting point in designing modern antennas, which is rarely attainable. In contrast, global optimization methods seem to attract more interest because they are robust and require no significant modifications during their design or implementation. However, they usually necessitate a significant number of electromagnetic simulations, which can be costly, to obtain optimal design parameters [35]. A particularly efficient alternative to conventional methods for resolving these issues is metaheuristic optimization. Meta-heuristic optimization algorithms, unlike other optimization techniques, are generally able to avoid local optima and are practical for a wide range of applications and domains without requiring significant changes to their implementation and design. This makes them superior to other optimization techniques. However, metaheuristic algorithms have limitations.

The Chimp Optimization Algorithm (ChOA)'s inspiration is drawn from the intelligence and sexual drive of chimpanzees. The ChOA algorithm exhibits adaptability in adjusting exploration and exploitation, demonstrating a commendable convergence speed. However, it possesses certain limitations such as constrained exploration capacity, susceptibility to local optima, and unsuitability for discrete problems. Xiang et al. proposed an improved ChOA algorithm that introduces a range of improvements including the implementation of the quantum coding method, by employing the quantum coding method, the population initialization stage witnesses an augmented diversity. Similarly, the iterative process benefits from the multiple-population strategy, which enhances the diversity of solutions in search of superior outcomes. Genetic operators strengthen the algorithm's exploration capability, while the local search strategy aids in the pursuit of better solutions within the vicinity of each existing solution. The improved ChOA locates solutions more quickly and accurately [36]. The Artificial Bee Colony (ABC), is built by observing how bees locate food and communicate information about food sources to the bees in the nest. Despite its strong exploration ability, ABC shows a relatively slow convergence rate and a low population diversity. To tackle these shortcomings, Zhou et al. proposed a novel ABC with Multiple Neighborhood Topologies (ABC-MNT). ABC-MNT assigns three distinct topologies to individuals, enabling dissimilar capacities in propagating information across individuals and achieving a smoother balance between exploration and exploitation. Additionally, to retain the experience gained during the search, global opposition-based learning and neighborhood search methods are employed. Experimental results confirmed that ABC-MNT outperforms or at least achieves similar performance on most benchmark functions [37]. The Particle Swarm Optimization (PSO) mimics social animal habits, such as school of fish, and bird flocking, by utilizing the swarm theory. It also has early convergence, especially in complex multi-peak search problems. To overcome these limitations, Wang et al. proposed a hybrid PSO algorithm that incorporates an Adaptive Learning Strategy, Adaptive Learning strategy Particle Swarm Optimization (ALPSO). Within ALPSO, a self-learning-based candidate is employed in the generation strategy to enhance the algorithm's exploration capability, while a competitive learning-based prediction strategy ensures effective exploitation. To strike a balance between exploration and exploitation, a tolerance-based search direction adjustment mechanism was devised. Experimental results showcase the superior performance of ALPSO compared to other algorithms in a wide range of cases [38].

To summarize, the ChOA algorithm prioritizes adaptability but faces challenges such as limited exploration capacity, vulnerability to local optima, and unsuitability for discrete problems. However, an enhanced version of ChOA has been developed to improve its search capabilities. Unlike ChOA, the ABC algorithm emphasizes exploration but suffers from slow convergence and low diversity. The introduction of ABC-MNT addresses these issues and demonstrates good performance on benchmark functions. Similar to ABC, the PSO algorithm tends to converge early but lacks exploration ability. However, the hybrid PSO algorithm with ALPSO addresses this limitation and shows superior performance in various scenarios. In conclusion, each of these algorithms has its own limitations that call for further improvement.

This paper focuses on HBA introduced by Hashim et al. [14]. The HBA is a metaheuristic algorithm that draws inspiration from honey badger foraging behavior. HBA is very stable, easy to use, and has a clear structure. But like other metaheuristic algorithms, the HBA has drawbacks such as a limited ability for global exploration, a slow convergence rate, poor precision, and a propensity to settle for a local optimum. These limitations corroborate the No-Free-Lunch theorem (NFL) which demonstrates logically that there is no one optimization algorithm that is applicable to resolve all types of optimization problems [39,40]. Consequently, this work introduces a Gold Sine mechanism-based Honey Badger Algorithm with Tent chaos (GST-HBA), to tackle the aforementioned challenges in the original HBA. The new GST-HBA is applied to the antenna S-parameter optimization problem.

The rest of this work is organized as follows: Section 2 proposes an intense review of some well-known meta-heuristic optimization algorithms for antennas and Section 3 explains the original honey badger algorithm in detail. Section 4 describes the antenna S-parameter optimization problem. Section 5 presents the proposed GST-HBA; the performance of the GST-HBA is analyzed through experimental simulation and the results shown in section 6, in Section 7, GST-HBA is applied to antenna S-parameter optimization problem; Section 8 discusses the uncertainties and limitations associated with antenna S-parameter optimization. Section 9 explains the conclusion and future work.

## 2 Literature review

This section provides a review of several algorithms that have been improved by researchers to address antenna optimization problems. These algorithms aim to enhance the performance of different types of antennas by optimizing various features and overcoming specific challenges as expressed in Table 1 below.

**Table 1:** Meta-heuristic optimization algorithms for antenna problems.

Author names and citation	Algorithm	Challenges	Type of antenna problem	Method
Subhashini and Satapathy [41]	Enhanced Ant Lion Optimization (e-ALO)	Find the best arrangement (spacing) of antenna array elements and their excitations for various antenna shapes, with the goal of minimizing sidelobe levels while remaining within boundary limitations for other restrictions.	Antenna array	Rather than using a uniform probability distribution function, the Pareto distribution was chosen from several other distributions and used to obtain a random number in the interval [0, 1]. Additional weighting variables were also incorporated during the ant location updating stage, bringing their movements near both the Roulette wheel and the elite individual.
Guttula et al. [32]	Elephant Herding Optimization with New Scaling Factor (EHO-NSF)	Improve narrow bandwidth which is not suitable for broadband equipment.	Microstrip Patch Antennas (MPA)	A new scaling factor is introduced for better search quality. The modification resulted in improving the antenna gain by choosing optimal values for the width, the length of the patch, the thickness, and the dielectric value of the substrate of the MPA.
Janairo et al. [42]	Genetic Programming with Lichtenberg Algorithm (GP-LA), Genetic Programming with Henry Gas Solubility Optimization (GP-HGSO), and Genetic Programming with Archimedes Optimization Algorithm (GP-AOA)	Capacitance improvement	Plate-wire antenna	In order to establish quasi-static conditions, the antenna capacitance fitness function was constructed using GP and then minimized using GP-LA, GP-HGSO, and GP-AOA. Finally, using Altair Feko, the three antennas were 3D designed, then their electrical characteristics were compared to those of the standard antenna. The hybrid GP-LA antenna model produced practical outputs, while the hybrid GP-AOA and GP-HGSO produced coupled transceiver systems with inappropriate antenna feature capacitance.
Li et al. [43]	Neighborhood-Redispatch Particle Swarm Optimization (NR-PSO)	Design and optimize a compact Ultrawideband (UWB) antenna that does not require an additional resonance structure for Bluetooth applications with a Wireless Local Area Network (WLAN) stopband	UWB antenna	The proposed algorithm includes three new factors: convergence factor, neighborhood factor, and dispatch factor. The first method is utilized to determine if a particle has entered the convergence area centered on the present global optimal. The second specifies the neighborhood research space into which a particle from the convergence decision area is redirected. The third component specifies the number of particles that will be redirected to the nearby research area. The three components combine to form a successful system in PSO, resulting in strong optimization performance.
Singh and Kaur [44]	Levy flight Archimedes Optimizer (LAO)	Overcome the limitations of the original Archimedes optimizer, namely its tendency to converge too slowly and prematurely, which causes it to be stuck in local optima	Microstrip Patch antenna	A fixed limit is set for every decision variable; if a variable fails to reach its optimal solution within the search area till the end of the current generation, the limit for that variable is adjusted. If a decision variable exceeds the boundary, the levy flight introduction helps to restrict its speed hence improving the exploration phase. The Levy flight is also utilized to compute the step size for random walks and ignore the Archimedes Optimizer Algorithm's local optima while searching.
Pal et al. [45]	Modified Invasive Weed Optimization (M-IWO)	Design of time-modulated linear antenna arrays with ultra-low Side Lobe Level (SLL), Side Band Level (SBL), and Main Lobe Beam Width often specified as Beam Width First Null (BWFN)	Linear antenna	Two parallel populations and a more explorative approach that involved altering the mutation step size over time were introduced to the original algorithm.

Table 1. Shows each algorithm's main challenge addressed, the type of antenna problem targeted, and the method applied. In summary, these algorithms address different challenges in antenna design, such as selecting significant features, minimizing side lobe levels, improving narrow bandwidth, enhancing capacitance, achieving compact designs, overcoming convergence limitations, and optimizing various antenna parameters.

## 3 Honey badger Algorithm (HBA)

Recently, researchers introduced the HBA, a nature-inspired algorithm inspired by the intelligent foraging behavior of honey badgers [14]. The honey badger employs two main hunting techniques: following “honeyguide birds and using its olfactory” abilities to search for food sources. In the exploration phase of the algorithm, inspired by both strategies, the honey badger locates its prey by relying on its sense of smell. Once it identifies the prey, the honey badger explores the surrounding area to find the optimal spot for capturing the prey. In the exploitation phase, inspired by the second hunting technique, the honey badger track food source with the aid of honeyguide birds to reach the hive. Its pace is influenced by the intensity of the prey scent detected at its current location, facilitating efficient exploitation.

First step: Initialization.

Each potential solution's position is shown as a vector in the D dimension, with the formula $x_{i}=\left[x_{i}^{1},x_{i}^{2},\ldots ,x_{i}^{D}\right]$. The mathematical relation below is utilized to initialize the different positions of honey badgers with an n population:

(1) $$x_{i}=lb_{i}+r_{n1}\times \left(ub_{i}-lb_{i}\right)$$

where $ub_{i}$ is the search space maximum limit, $lb_{i}$ denote the search space minimum limit, $x_{i}$ is a potential solution in a population of size N. $r_{n1}$ is a number selected randomly within a range of [0, 1].

Second Step: Intensity definition.

The following equation presents the relation between the scent intensity $I_{i}$, the concentration strength S, and the distance $d_{i}$ between the honey badger and the prey.

(2) $$\begin{array}{c} I_{i}=r_{n2}\times \frac{S}{4\pi d_{i}^{2}}\\ S={\left(x_{i}-x_{i+1}\right)}^{2}\\ d_{i}=x_{prey}-x_{i} \end{array}$$

where $x_{i}$ denotes honey badger's current position, $x_{i+1}$ represents honey badger's immediate next position, $x_{prey}$ denotes the prey position, while $r_{n2}$ represents a selected arbitrary number from the range [0, 1].

Third Step: Updating density factor.

(α), the density factor is a randomization regulator employed to obtain an appropriate balance between the exploitation and exploration phases. α lowers with iterations to minimize population variety.

(3) $$\alpha =C\times \exp\left(\frac{-\text{it}}{{it}_{\max}}\right)$$

C represents a number drawn arbitrarily from the interval [1, +∞]. it is the current iteration, while ${it}_{\max}$ represent maximum iteration number.

Fourth Step: Local optimum escape and updating the position of the agents.

This action is used to limit the risks of being trapped in the local optimum. The HBA generates a flag Fl to alter the direction of the search in a bid to enhance the possibilities of a thorough scan of the search area by the agents. The “honey phase” as well as the “digging phase” are the two subphases of the updating process for the HBA position. They are detailed as follows:

Fifth Step Digging phase: During this particular phase, the behaviors exhibited by the honey badger are expressed as:

(4) $$x_{\text{new}}=x_{\text{prey}}+Fl\times \beta \times I\times x_{\text{prey}}+Fl\times r_{n3}\times \alpha \times d_{i}\times [\cos\left(2\pi r_{n4}\right)\times \left[1-\cos\left(2\pi r_{n5}\right)\right]$$

$x_{\text{prey}}$ denotes the prey's position and the global best position, β represents the honey badger ability, it's given as a preset value ≥ 1 (initial value = 6), while $r_{n3}$, $r_{n4}$, and $r_{n5}$ are three distinct numbers drawn arbitrarily from the interval [0, 1]. Fl is a flag that changes the search's direction and is calculated using Eq. (5):

(5) $$Fl=\left\{\begin{array}{c} 1\text{if}\;r_{n6}\leq 0.5\\ -1\text{else,} \end{array}\right\}$$

$r_{n6}$ is a randomly selected number from [0, 1]. The intensity of the prey's scent, the badger's position, and the parameter α affect prey detection. Disruption Fl may be encountered in the digging processes.

Honey phase**:** The instance at which the honey badger follows the honeyguide to get to the beehive.

(6) $$x_{\text{new}}=x_{\text{prey}}+Fl\times r_{n7}\times \alpha \times d_{i}$$

where $r_{n7}$ and $x_{\text{new}}$ denote a random digit within [0,1] and a new position for the badger.

**Fig. 1:**
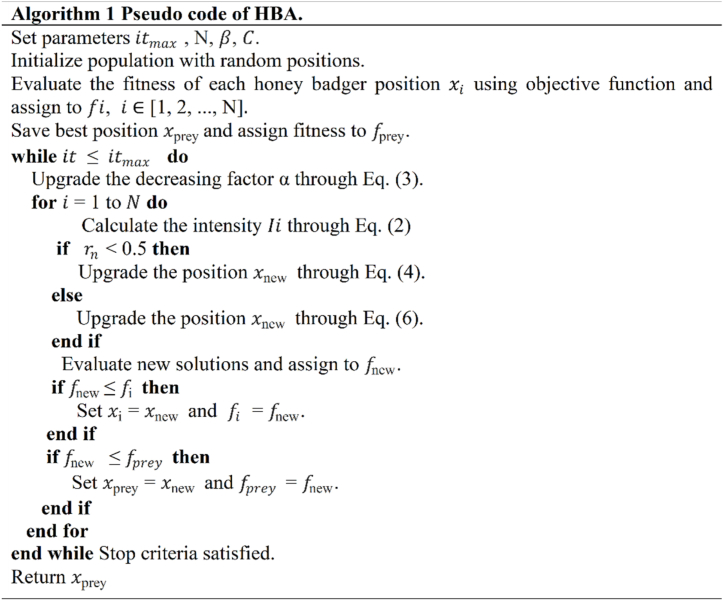
HBA pseudo code.

The pseudo code of HBA is given in Fig. 1.

## 4 Antenna S-parameter optimization problem

In the optimization algorithms’ preliminary stage of design, they are usually evaluated on a collection of analytical functions for which the global and local optima are known. This collection of functions, also known as a test suite helps in validating the performance of the algorithm in relation to its effectiveness and efficiency in numerous circumstances [46,47]. Several authors have proposed test suites for evaluating the effectiveness of optimization methods function when applied to antenna problems. One such test suite is by Ref. [48]. They presented a set of functions that may be used to gauge and assess how well various Evolutionary optimization Algorithms (EAs) perform when used to address difficult Electro-Magnetic (EM) challenges. Liu et al. have also proposed a test suite to simulate electromagnetic problems for filter design [49]. Recently introduced by Zhang et al. is a test set of eight functions [50]. In the proposed set, each function corresponds to a particular landscape of the “EM simulated antenna problems”. Antenna landscapes depict the projected three-dimensional correlation between the values of design parameters and the objective function in the context of antenna problems. They serve as visual representations to showcase the optimization properties of antennas. By aligning the characteristics of a test function with an antenna landscape, one can effectively evaluate the performance of an antenna optimization algorithm.

### 4.1 Several types of antenna structures

It is nearly impossible to look into every type of antenna that has been developed over the years due to the enormous quantity of them. Based on their structural characteristics, antennas are categorized into four groups as presented in the following subsections [50].

#### 4.1.1 Single-antenna with single-band

Considered the simplest antenna problem, it refers to single antennas that transmit and receive data on a single frequency band. The system can only transmit or receive one data stream at a time, and it can only operate on one frequency band at a time. Some examples are monopole and dipole antennas. Typically, theoretical calculations can be used to determine the resonance frequency of this type of antenna.

#### 4.1.2 Single-antenna with multi-band

It refers to systems that use a single antenna to transmit and receive data on multiple frequency bands. Meaning the system can transmit and receive data on different frequency bands. This sort of antenna is built with parasitic components that have various resonance frequencies in order to increase bandwidth or add more bands.

#### 4.1.3 Multi-antenna with one feeding

It refers to multiple antennas that transmit and receive data but with only one feed line connecting the antennas to the transceiver. The antennas are physically combined into a single structure or array, which allows them to share a common feed point. This type of antenna might result in a multi-objective optimization problem. It is possible to employ resonant frequency, S-parameters, and any pattern-related parameters, such as axial ratio, gain, and polarization concurrently as objective functions.

#### 4.1.4 Multi-antenna with multi-feeding

It refers to systems that use multiple antennas and multiple feed lines for data transmission and reception. Each antenna is attached to a separate feed line, which allows each antenna to operate independently and transmit or receive data on its own. The feed lines are connected to a signal processing unit, which can combine or separate the signals from each antenna as needed to optimize performance. Each component might have specific polarization and directivity properties. To increase isolation, several polarization modes are occasionally used. With the aforementioned classification, A standard set of test functions was created for the antenna S-parameter, each function illustrates an antenna-type landscape [50]. The test suite is described in Table 2.

**Table 2:** S-parameter test functions.

Formula	Dimension	Antenna Type	F_min_
$A_{1}\left(x\right)=20\log\left(2\left(\sum_{i=1}^{n}\left|{\left(\sin\left(\frac{x_{i}}{8}\right)\right)}^{2}\right|+\prod_{i=1}^{n}\left|\sin\left(\frac{x_{i}}{8}\right)\right|\right)+1\right)$	8	Single	0
$A_{2}\left(x\right)=20\log\left(10{\left(\sum_{i=1}^{n}x_{i}^{2}\right)}^{2}+1\right)$	8	Multiple	0
$A_{3}\left(x\right)=20\log\left(10{\left(\sum_{i=1}^{n}0.01i^{5}x_{i}^{2}\right)}^{2}+1\right)$	8	Single and Multiple	0
$A_{4}\left(x\right)=20\log\left(\left(\sum_{i=1}^{n-1}\left(100{\left(x_{i+1}-x_{i}^{2}\right)}^{2}-{\left(x_{i}-1\right)}^{2}\right)\right)+1\right)$	8	Multiple	0
$A_{5}\left(x\right)=100\sqrt{\left|x_{2}+1-0.01{\left(x_{1}-10\right)}^{2}\right|}+0.01\left|x_{1}\right|$	2	Multiple	0
$A_{6}\left(x\right)=20\log\left(0.01{\left(\sum_{i=1}^{n}\left|x_{i}\right|\right)}^{2}{\left(\sin\left(0.8x_{1}\right)+2\right)}^{4}+1\right)$	8	Multiple	0
$\begin{array}{c} A_{7}\left(x\right)=20\log\left(\left(\sum_{i=1}^{n-1}\left(100{\left(x_{i+1}-x_{i}^{2}\right)}^{2}-{\left(x_{i}-1\right)}^{2}\right)\right)+1\right)\\ +20\log\left(0.01{\left(\sum_{i=1}^{n}\left|x_{i}\right|\right)}^{2}{\left(\sin\left(0.8x_{1}\right)+2\right)}^{4}+1\right) \end{array}$	8	Multiple	0
$\begin{array}{c} A_{8}\left(x\right)=100\sqrt{\left|x_{2}+1-0.01{\left(x_{1}-10\right)}^{2}\right|}+0.01\left|x_{1}\right|\\ +20\log\left(0.01{\left(\sum_{i=1}^{n}\left|x_{i}\right|\right)}^{2}{\left(\sin\left(0.8x_{1}\right)+2\right)}^{4}+1\right) \end{array}$	8	Multiple	0

## 5 Golden sine mechanism-based honey badger algorithm with tent chaos (GST-HBA)

### 5.1 Tent chaos mechanism

**Fig. 2:**
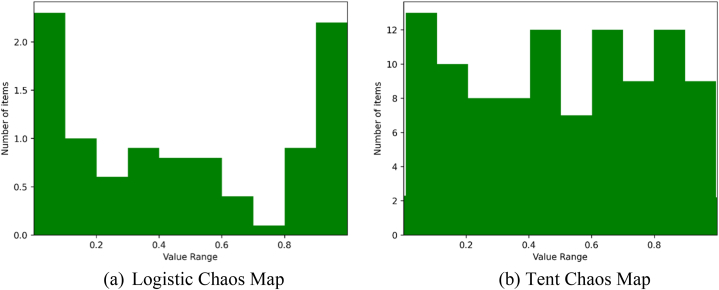
Sequence map.

Chaos mapping is a sophisticated dynamic technique used in nonlinear systems that exhibits randomness, ergodicity, and regularity. It is applied widely for algorithm optimization to achieve a more thorough and extensive exploration of the search space. Currently, Tent Chaotic mapping (TC) and Logistic Chaotic mapping (LC) are the two most popular chaos. Demir et al. contrasted the impacts of LC with TC [51]. They were able to conclude that TC shows a more uniform population distribution and results in faster convergence. As displayed by Fig. 2a, the outputs produced by LC between [0.0, 0.2] and [0.8, 0.1] are bigger than those of other sections, while Fig. 2b shows that TC values are more uniform over all viable locations. Based on these, TC is widely adopted to substitute the random initialization of algorithms in order to ensure higher variety in the starting population, better convergence speed, and reduce the algorithm's tendency to be trapped in a local optimum.

TC can be addressed by the following equation

(7) $$x_{i+1}=\left\{ \begin{array}{c} 2x_{i},0\leq x\leq 0.5\\ 2\left(1-x_{i}\right),0.5<x\leq 1 \end{array}\right.$$

$x_{i}$ is the decision variable of a potential solution.

Eq. (8) is the Bernoulli shift transformed version of the previous equation:

(8) $$x_{i+1}=\left(2x_{i}\right)\mathrm{mod}\;1$$

To avoid the possibilities of the TC expression entering short and unstable period points throughout the iteration process, and to retain its regularity, ergodicity, and unpredictability, $\text{rand}\left(0,1\right)\times \frac{1}{N}$, is a random variable incorporated as presented in Eq. (9):

(9) $$x_{i+1}=\left\{ \begin{array}{c} 2x_{i}+\text{rand}\left(0,1\right)\times \frac{1}{N},0\leq x\leq 0.5\\ 2\left(1-x_{i}\right)+\text{rand}\left(0,1\right)\times \frac{1}{N},0.5<x\leq 1 \end{array}\right.$$

Transformed by the Bernoulli shift, it is expressed as:

(10) $$x_{i+1}=\left(2x_{i}\right)\mathrm{mod}\;1+\text{rand}\left(0,1\right)\times \frac{1}{N}$$

where rand⁡(0,1) represents a number drawn arbitrarily from the interval [0, 1], and N denotes the total number of individuals in the sequence.

### 5.2 Golden sine (GS) mechanism

**Fig. 3:**
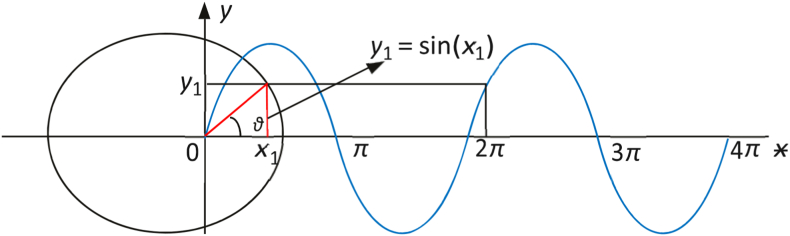
Relationship between the unit circle and the sine function.

**Fig. 4:**
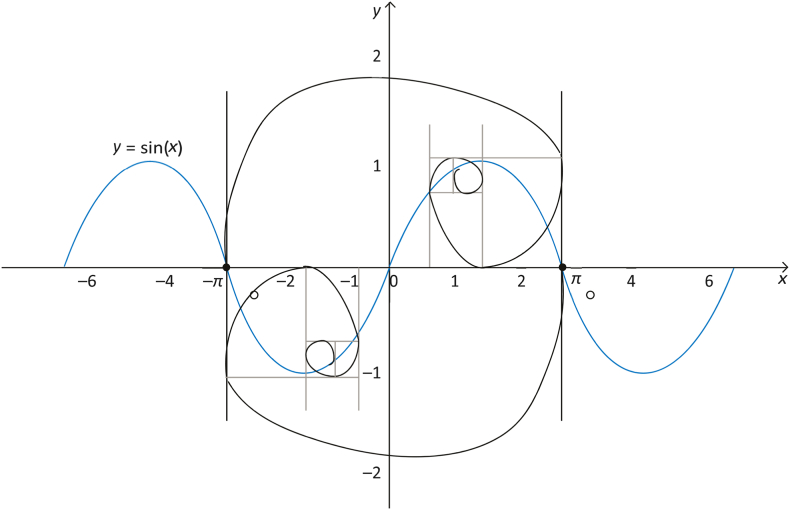
Solution of GS mechanism.

The GS algorithm which was proposed by Tanyildizi et al. is a new metaheuristic algorithm that simulates the golden ratio and the mathematical sine function [52]. The GS algorithm performs an iterative search to approach the optimal solution by combining a sine function and the golden ratio. As illustrated in Fig. 3, the sine curve has a unique connection with the unit circle and is defined inside the interval [−1, 1] with a period of 2π. The sine function's dependent variable changes when the related independent variable's value changes. In other terms, exploring all the positions on the unit circle is equal to exploring all sine function values. Based on this principle, the space for the search is progressively narrowed and the search is carried out in areas where there is a greater likelihood of reaching the optimal solution to increase efficiency of convergence [53], the model of the GS algorithm population update method is illustrated in Fig. 4.

After updating the population, the best potential solution is assessed in the population, and its position is modified using GS as follows:

(11) $$x_{\text{new}}=x*\left|\sin\left(r_{1}\right)\right|-r_{2}*\sin\left(r_{1}\right)*\left|c_{1}*x_{\text{best}}-c_{2}*x\right|$$

where $x_{\text{best}}$ is the best potential solution globally, x denotes an individual's current solution, $c_{1}$ and $c_{2}$ represent the coefficient factors, $r_{1}$ is a number taken arbitrarily from [0, 2π] and $r_{2}$ is a number selected arbitrarily from the interval [0, π ].

(12) $$c_{1}=a*\left(1-\tau \right)+b*\tau$$

(13) $$c_{2}=a*\tau +b*\left(1-\tau \right)$$

The Golden ratio is τ= (1 + 1)/2, while a and b are the golden ratio's initial values (they are problem dependent). The generated individual using GS must then be compared to the ideal solution, coefficient factors $c_{1}$ and $c_{2}$ are adjusted based on the result from the comparison. The new individual created using GS is compared to the optimal, and the coefficient factors $c_{1}$ and $c_{2}$ are modified accordingly.

If $f\left(x_{\text{new}}\right)<f\left(x_{\text{best}}\right)$.

(14) $$b=c_{2},c_{2}=c_{1},c_{1}=a*\tau +b*\left(1-\tau \right)$$

Else

(15) $$b=c_{1},c_{1}=c_{2},c_{2}=a*\left(1-\tau \right)+b*\tau$$

In case $c_{1}=c_{2}$ the variables are computed as follows

(16) a=rand⁡(0,π)

(17) b=rand⁡(0,−π)

(18) $$c_{1}=a*\tau +b*\left(1-\tau \right),c_{2}=a*\left(1-\tau \right)+b*\tau$$

Using GS to enable the best candidate's values in order to increasingly approach the optimal solution may result in a balance between local exploitation and global exploration.

### 5.3 Work flow of the proposed GST-HBA

**Fig. 5:**
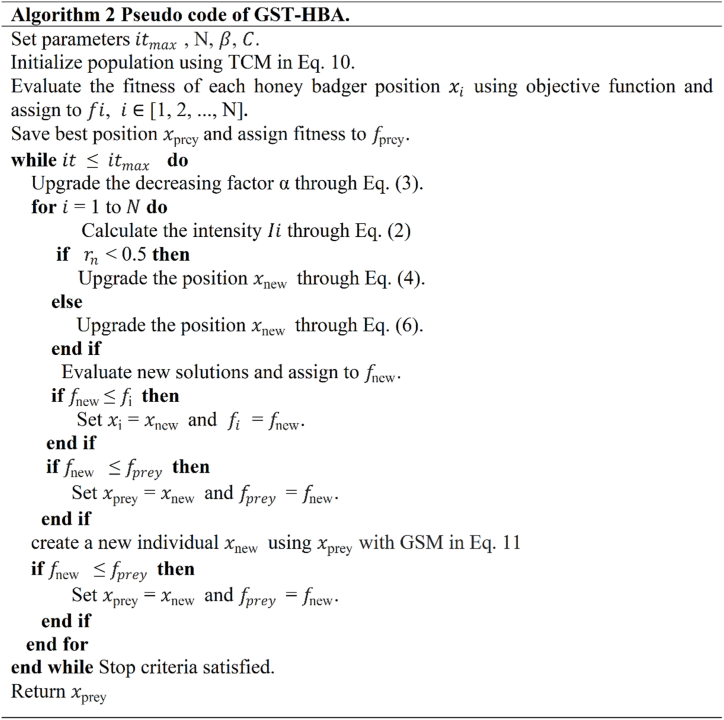
GST-HBA pseudocode.

The pseudocode for GST-HBA is outlined in Algorithm 2, presented in Fig. 5. The initial step in the optimization process involves populating the search space randomly. In GST-HBA, we utilize the TC approach for population initialization, which is expressed in Eq. (10) to enhance population diversity, as demonstrated in Algorithm 2. Once the population is initialized, we employ the core mechanism of HBA until Eq. (6), which is responsible for updating the position of each honey badger. Subsequently, GS expressed in Eq. (11) is introduced to generate a new position for the best honey badger in the population. If this new position proves to be superior to the current position, it is used to update the position of the best honey badger, thereby further enhancing its solution. Iterations proceed until the maximum number of iterations is reached, signaling the termination of the algorithm.

## 6 Simulation experiment and result analysis

The Honey Badger Algorithm (HBA) [14], Differential Evolution (DE) [54], Jaya Optimization Algorithm (JAYA) [55], and Sine Cosine Algorithm (SCA) [56] were used as a control group and 20 groups of Congress on Evolutionary Computation (CEC) benchmark functions [6], commonly used by researchers, were used for testing in order to assess the GST-HBA's performance. Mono-peaked functions F1–F7, more challenging multimodal functions are F8–F13 with several peaks, and fixed dimensions multimodal functions are seen in F13–F20 as recorded in Table 3. Every algorithm was run 30 times separately, with 500 iterations per run, the population size is 30 and the dimension of function F1–F13 is 30 to ensure that the experiment is fair and to reduce mistakes. The Standard Deviation (STD), as well as the Average value (AVG) of the results of the 30 independent runs, are selected as evaluation metrics. The mean may be used to estimate the algorithms' stability, whereas the STD can be used to evaluate the accuracy of the algorithm, the findings are presented in Table 4. The parameters of each algorithm are set as explained in the original literature of each algorithm.

**Table 3:** Benchmark functions.

No	Function	Dimension	Range	Fmin
$F_{1}$	$f_{1}\left(x\right)=\sum_{i=1}^{n}\;x_{i}^{2}$	30	[−100,100]	0
$F_{2}$	$f_{2}\left(x\right)=\sum_{i=1}^{n}\;\left|x_{i}\right|+\prod_{i=1}^{n}\;\left|x_{i}\right|$	30	[−10,10] 0]	0
$F_{3}$	$f_{3}\left(x\right)=\sum_{i=1}^{n}\;{\left(\sum_{j-1}^{i}\;x_{j}\right)}^{2}$	30	[−100,100]	0
$F_{4}$	$f_{4}\left(x\right)=\min_{i}\;\left\{\left|x_{i}\right|,1\leq i\leq n\right\}$	30	[−100,100]	0
${\begin{array}{c} F \end{array}}_{5}$	$f_{5}\left(x\right)=\sum_{i=1}^{d}\sum_{j=1}^{i}x_{j}^{2}$	30	[−65.536,65.536]	0
$F_{6}$	$f_{6}\left(x\right)=\sum_{i=1}^{n}\;{\left(\left[x_{i}+0.5\right]\right)}^{2}$	30	[−100,100]	0
$F_{7}$	$f_{7}\left(x\right)=\sum_{i=1}^{n}\;ix_{i}^{4}+\text{random}[0,1)$	30	[−1.28,1.28]	0
$F_{8}$	$f_{8}\left(x\right)=1-\cos\left(2\pi \sqrt{\sum_{i=1}^{d}x_{i}^{2}}\right)+0.1\sqrt{\sum_{i=1}^{d}x_{i}^{2}}$	30	[−100,100]	0
$F_{9}$	$f_{9}\left(x\right)=\sum_{i=1}^{n}\;\left[x_{i}^{2}-10\cos\left(2\pi x_{i}\right)+10\right]$	30	[−5.12,5.12]	0
$F_{10}$	$f_{10}\left(x\right)=-20\exp\left(-0.2\sqrt{\frac{1}{n}\sum_{i=1}^{n}\;x_{i}^{2}}\right)-\exp\left(\left(1/n\right)\sum_{i=1}^{n}\;\cos\left(2\pi x_{i}\right)\right)+20+e$	30	[−32,32]	0
$F_{11}$	$f_{11}\left(x\right)=1/4000\sum_{i=1}^{n}\sum x_{i}^{2}-\prod_{i=1}^{n}\;\cos\left(x_{i}/\sqrt{i}\right)+1$	30	[−600,600]	0
$F_{12}$	$f_{12}\left(x\right)=\pi /n\left\{\sum_{i=1}^{n-1}\;{\left(y_{i}-1\right)}^{2}\left[1+10\sin^{2}\left(\pi y_{i+1}\right)\right]+{\left(y_{n}-1\right)}^{2}\right\}$ $+\sum_{i=1}^{n}\;u\left(x_{i},10,100,4\right)+\pi /n10\;\sin\left(\pi y_{1}\right)y_{i}=1+x_{i}+\left(1/4\right)u\left(x_{i},a,k,m\right)=\left\{ \begin{array}{ll} k{\left(x_{i}-a\right)}^{m} & x_{i}>a\\ 0 & -a<x_{i}<a\\ k{\left(-x_{i}-a\right)}^{m} & x_{i}<-a \end{array}\right.$	30	[−50,50]	0
$F_{13}$	$f_{13}\left(x\right)=0.1\left\{\sum_{i=1}^{n}\;{\left(x_{i}-1\right)}^{2}\left[1+\sin^{2}\left(3\pi x_{i}+1\right)\right]+{\left(x_{n}-1\right)}^{2}\left[1+\sin^{2}\left(2\pi x_{n}\right)\right]\right\}+0.1\sin^{2}\left(3\pi x_{1}\right)+\sum_{i=1}^{n}\;u\left(x_{i},5,100,4\right)$	30	[−50,50]	0
$F_{14}$	$f_{14}\left(x\right)=\left|x^{2}+y^{2}+xy\right|+\left|\sin\left(x\right)\right|+\left|\cos\left(y\right)\right|$	2	[−500,500]	1
$F_{15}$	$f_{15}\left(x\right)=\sin^{2}\left(3\pi x\right)+{\left(x-1\right)}^{2}\left(1+\sin^{2}\left(3\pi y\right)\right)+{\left(y-1\right)}^{2}\left(1+\sin^{2}\left(2\pi y\right)\right)$	4	[−10,10]	0
$F_{16}$	$f_{16}\left(x\right)=4x_{1}^{2}-2.1x_{1}^{4}+1/3x_{1}^{6}+x_{1}x_{2}-4x_{2}^{2}+4x_{2}^{4}$	2	[−5,5]	−1.0316
$F_{17}$	$f_{17}\left(x\right)={\left(x_{2}-5.1/4\pi^{2}x_{1}^{2}+5/\pi x_{1}-6\right)}^{2}+10\left(1-\left(1/8\pi \right)\right)\cos x_{1}+10$	2	[−5,5]	0.398
$F_{18}$	$f_{18}\left(x\right)=\left[1+{\left(x_{1}+x_{2}+1\right)}^{2}\left(19-14x_{1}+3x_{1}^{2}-14x_{2}+6x_{1}x_{2}+3x_{2}^{2}\right)\right]\times \left[30+{\left(2x_{1}-3x_{2}\right)}^{2}\times \left(18-32x_{1}+12x_{1}^{2}+48x_{2}-36x_{1}x_{2}+27x_{2}^{2}\right)\right]$	2	[−2,2]	3
$F_{19}$	$f_{19}\left(x\right)=-\sum_{i=1}^{4}\;c_{i}\;\exp\left[-\sum_{j=1}^{3}\;a_{ij}{\left(x_{j}-p_{ij}\right)}^{2}\right]$	3	[1,3]	−3.86
$F_{20}$	$f_{20}\left(x\right)=x^{2}+2y^{2}-0.3\cos\left(3\pi x\right)\cos\left(4\pi y\right)+0.3$	2	[−100,100]	0

**Table 4:** Comparison of GST-HBA with other algorithms.

		GST-HBA	HBA	DE	JAYA	SCA
F1	AVG	**1.033E-277**	5.951E-29	1.627E-4	1.039E-4	9.250E+0
	STD	**0**	3.205E-28	1.277E-4	2.361E-4	0
F2	AVG	**8.517E-141**	7.044E-18	4.973E-3	3.217E-4	2.223E-2
	STD	**0**	2.051E-18	1.493E-3	5.093E-5	1.897E-2
F3	AVG	**7.672E-259**	1.646E-9	2.143E+4	2.064E+4	8.909E+3
	STD	**0**	6.159E-12	2.142E+3	5.732E+3	5.324E+3
F4	AVG	**2.660E-141**	2.700E-11	1.280E+1	2.029E+1	3.680E+1
	STD	**2.369E-147**	4.600E-11	2.118E+0	4.417E+0	1.193E+0
F5	AVG	**4.438E-276**	6.867E-24	6.739E-4	2.549E-4	8.136E+1
	STD	**0**	4.813E-26	1.184E-4	1.164E-4	1.073E+1
F6	AVG	1.748E-2	6.926E-2	**1.598E-4**	3.869E+0	3.553E+1
	STD	2.059E-4	6.230E-3	**1.361E-5**	4.786E-1	6.259E-1
F7	AVG	**7.657E-4**	5.650E-3	4.185E-2	5.267E-2	8.314E-2
	STD	**1.693E-4**	3.108E-3	9.125E-3	2.911E-2	3.854E-4
F8	AVG	**4.047E-122**	1.029E-1	6.700E-1	9.483E-1	1.423E+0
	STD	**4.288E-127**	4.708E-2	8.897E-3	5.575E-4	1.267E+0
F9	AVG	**0**	7.031E+0	1.826E+2	9.779E+1	4.234E+1
	STD	**0**	3.406E+0	1.178E+1	4.389E+0	1.262E+1
F10	AVG	**4.441E-16**	1.015E+1	4.429E-3	3.446E-3	1.406E+1
	STD	**0**	6.782E-1	2.183E-3	6.921E-4	9.612E+0
F11	AVG	**0**	7.891E-4	2.255E-2	7.406E-2	8.961E-1
	STD	**0**	0	4.185E-2	3.206E-6	3.089E-2
F12	AVG	**3.229E-4**	1.035E-2	1.260E-2	1.244E+0	3.229E+4
	STD	**3.603E-5**	7.649E-5	4.974E-5	4.353E-1	1.548E+4
F13	AVG	4.521E-1	5.660E-1	**5.407E-3**	4.156E+2	5.778E+4
	STD	3.643E-1	5.725E-1	**5.793E-5**	1.855E+2	9.001E+1
F14	AVG	**1.000E+0**	**1.000E+0**	**1.000E+0**	2.291E+1	**1.000E+0**
	STD	**0**	**0**	**0**	1.027E-1	**0**
F15	AVG	**1.350E-31**	**1.350E-31**	**1.350E-31**	1.527E-2	1.772E-3
	STD	**0**	**0**	**0**	5.573E-4	5.982E-5
F16	AVG	**−1.032E+0**	**−1.032E+0**	**−1.032E+0**	**−1.032E+0**	**−1.032E+0**
	STD	**4.441E-16**	**4.441E-16**	**4.441E-16**	1.801E-4	4.561E-5
F17	AVG	**3.979E-1**	**3.979E-1**	**3.996E-1**	4.021E-1	4.003E-1
	STD	**0**	**0**	**0**	2.819E-3	2.633E-3
F18	AVG	**3.000E+0**	**3.000E+0**	**3.000E+0**	3.168E+0	**3.000E+0**
	STD	**0**	**0**	**0**	7.129E-1	1.043E-4
F19	AVG	**−3.863E+0**	**−3.863E+0**	**−3.863E+0**	−3.613E+0	−3.854E+0
	STD	1.776E-15	1.776E-15	1.776E-15	2.149E-1	2.728E-3
F20	AVG	**0**	**0**	**0**	2.267E-1	**0**
	STD	**0**	**0**	**0**	**0**	**0**
	**(+|-| = )**	(11|2|7)	(0|13|7)	(2|12|6)	(0|19|1)	(0|16|4)
	Friedmans Value	**1.55**	2.30	3.00	4.00	4.15
	Friedmans Rank	**1**	2	3	4	5

In Table 4, GST-HBA found the best near-ideal values for functions F1 through F6. In contrast to other state-of-the-art algorithms, it is better, but when compared to DE, it did not find the best value for function F7. This corroborates the NFL theorem as stated in the introduction, worthy of note is that compared to the original HBA there is a significant improvement in function F1–F7. This attests to the efficacy of the enhanced technique put forth in this paper. For F8-20, these functions test an algorithm's ability to explore the search space for the optimal solution since there are several local optimal solutions. For functions, F8 to F13 which are high-dimension functions, GST-HBA has the potential to find the optimum values for five functions, in functions F14 to F20 GST-HBA is able to obtain the theoretical optimal solution, in these functions, GST-HBA results are comparable to that of the Differential Evolution algorithm (DE), Honey Badger Algorithm (HBA) and Sine Cosine Algorithm (SCA) since they are fixed length multi-peaked functions that test the stability and exploration ability of an algorithm which means the improvement on HBA did not negatively impact the traditional techniques in the original HBA. It can be concluded from Table 4 that GST-HBA is also able to improve the population variety and escape local optimal value in the multi-modal functions, while in the single-modal function, it's able to explore regions for an ideal solution by employing the combination of TC and GS. The result also shows that there is a good tradeoff between the exploration ability of GST-HBA and its exploitation ability. For the proposed GST-HBA and the compared algorithms, we outlined in Table 4 the instances in which each algorithm achieved the best optimization value when compared to others, which is denoted by "+," the instances in which each algorithm did not identify the optimal solution, which is denoted by "-," and the instances in which each algorithm achieved results that were similar to those of the compared algorithms is denoted by “ = ”. Furthermore, in Table 4, the best AVG and STD value obtained for each function is highlighted in boldface.

### 6.1 Statistical test

As stated by Garcia [57] Optimization algorithms cannot be evaluated by mean and standard deviation values alone, in this research two popular nonparametric statistical tests are used to further evaluate the improvement of GST-HBA. Firstly, the Wilcoxon test is employed, if the Wilcoxon probability value of assumption also known P-value is 0.05 or greater, negate the null hypothesis, which would imply that the compared algorithms have no statistically significant differences. If the P-value is less than 0.05, accept the null hypothesis, which would mean there are substantial differences between the compared methods. In Table 5, the symbols "+", "-", and " = " denote that GST-HBA is excellent, inadequate, and similar, in comparison to the compared algorithms respectively. The P-values in Table 5 are all less than 0.05. Another statistical test that ranks the performance of methods is Friedmans test, which contrasts at least three matched or paired methods. Each algorithm's fitness value is ranked by the Friedman test from low to high [58], to elucidate the statistical improvement and distinction achieved by GST-HBA, we employ a Friedmans test. Leveraging the data presented in Table 4, Table 6, the Friedmans Value (FV) obtained through Equation (19) is utilized, this value indicates significant improvement of an algorithm in relation to its compared counterparts. Subsequently, the Friedman Rank (FR) for each optimizer is ascertained by arranging the average FV obtained for all functions in ascending order, with the most favorable outcome corresponding to the lowest value.

(19) $$\text{FV}=\frac{12\times n}{k\times \left(k+1\right)}\left[\sum_{j}\;R_{j}^{2}-\frac{k\times {\left(k+1\right)}^{2}}{4}\right]$$

in Eq. (19), the variables k, n, and $R_{j}^{2}$ correspondingly denote the count of algorithms, the count of benchmark functions, and the average result from benchmark functions associated with the j-th algorithm. Algorithms are assigned an FV on a spectrum ranging from 1 (indicating the most favorable result) to k (indicating the least favorable outcome). As seen in Table 4 GST-HBA ranked number one affirming it's significantly different from other algorithms

**Table 5:** Wilcoxon test.

GST-HBA vs	–	+	=	R-	R+	P-value
HBA	0	13	7	0	91	1.47E-03
DE	2	12	6	17	88	2.58E-02
JAYA	0	19	1	0	190	1.32E-04
SCA	0	16	4	0	136	4.38E-04

**Table 6:** Antenna S-parameter problem.

		GST-HBA	HBA	DE	JAYA	SCA
A1	AVG	**2.008E-2**	1.686E+0	1.692E+0	5.207E+0	1.380E+1
	STD	**0**	6.088E-2	3.761E-2	1.416E+0	8.335E-1
A2	AVG	**0**	**0**	1.088E+1	2.156E-5	5.523E+1
	STD	**0**	**0**	9.393E+0	2.067E+1	1.690E+1
A3	AVG	**0**	**0**	1.042E+2	2.142E+1	1.606E+2
	STD	**0**	**0**	8.297E+1	2.704E+1	4.080E+1
A4	AVG	**8.830E-6**	4.239E-5	7.851E+1	5.400E+1	1.287E+2
	STD	**3.291E-5**	1.575E-4	3.627E+1	3.636E+1	2.005E+1
A5	AVG	**1.953E-1**	6.472E-1	4.407E-1	3.886E+0	3.778E+0
	STD	**2.451E-2**	2.910E-1	2.603E-1	3.532E+0	3.604E+0
A6	AVG	**0**	4.106E-1	1.012E+1	2.666E+1	1.937E+1
	STD	**0**	4.247E-1	3.497E+0	1.320E+1	1.253E+1
A7	AVG	**3.084E+0**	3.178E+0	4.798E+1	1.448E+2	1.626E+2
	STD	**7.232E-1**	9.653E-1	4.928E+1	5.109E+1	3.325E+1
A8	AVG	**9.555E-90**	6.748E+1	3.230E+1	9.018E+1	8.907E+1
	STD	**5.146E-89**	1.884E+1	1.603E+1	1.839E+1	2.209E+1
	**(+|-| = )**	(6|0|2)	(0|6|2)	(0|8|0)	(0|8|0)	(0|8|0)
	Friedmans Value	**1.13**	2.19	3.06	4.00	4.63
	Friedmans Rank	**1**	2	3	4	5

### 6.2 Comparison of convergence curve

**Fig. 6:**
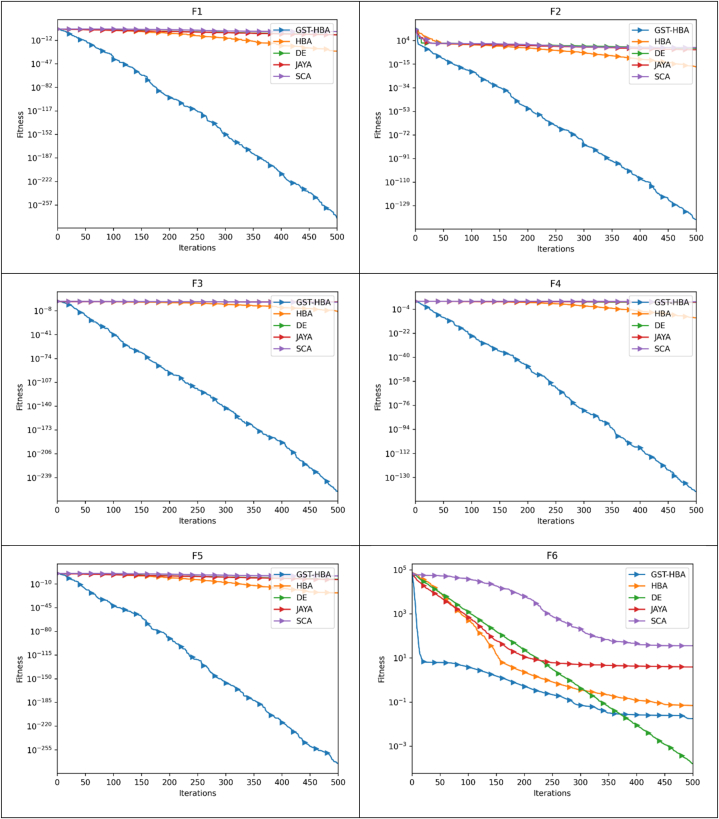

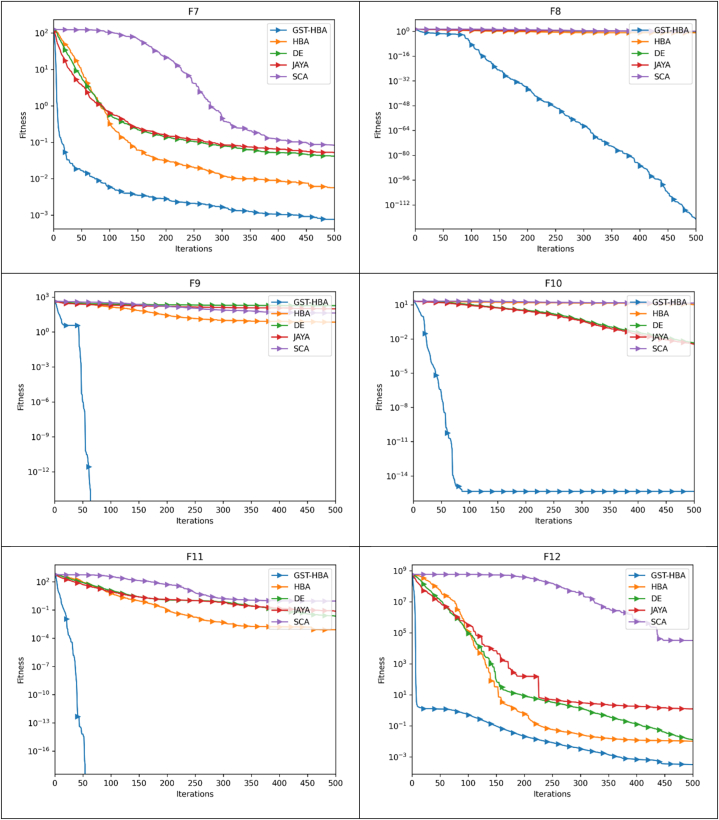

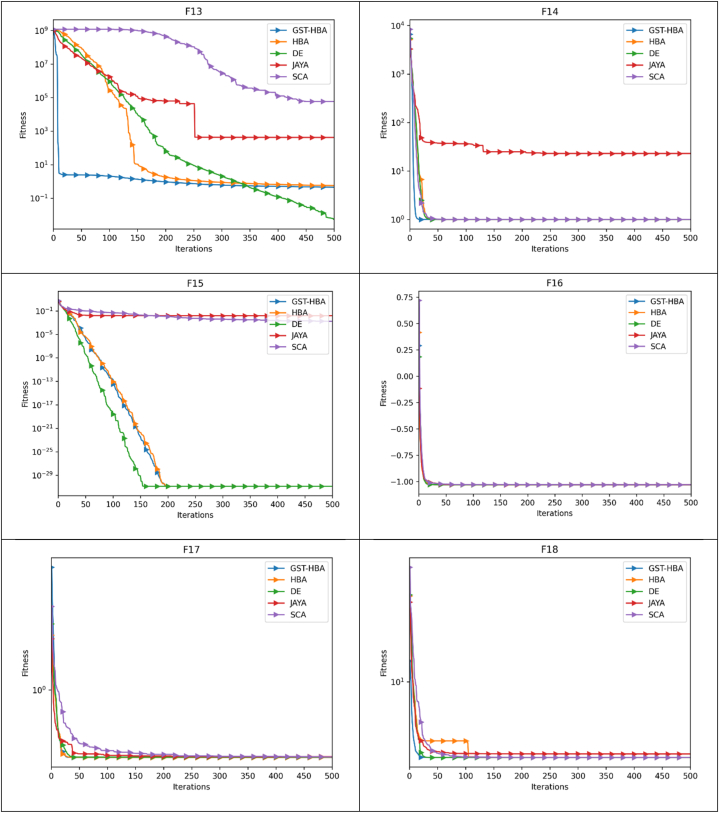

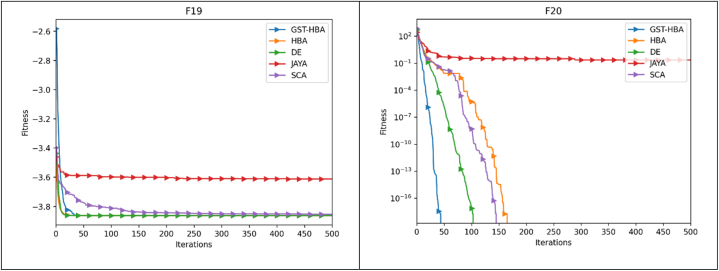
Convergence curve.

The optimization pace and precision may be more clearly shown by the convergence trajectory. The trajectory of convergence pertains to the path taken by the algorithm as it progressively moves toward the optimal solution during its iterative process. It showcases the algorithm's advancement towards achieving the most favorable result as iterations elapse. The speed of optimization, or optimization pace, denotes how swiftly the algorithm reaches the optimal solution. A rapid optimization pace signifies that the algorithm converges quickly, necessitating fewer iterations or steps to arrive at the optimal solution. Conversely, a slow optimization pace indicates that the algorithm requires more time and iterations to converge. The accuracy of the optimization algorithm signifies its ability to closely approach the true optimal solution. A highly precise algorithm will closely approximate the best possible outcome, while a less precise algorithm may yield a solution that deviates further from the optimal value. By analyzing the convergence trajectory, valuable insights can be gained regarding both the pace and precision of the optimization algorithm. The benchmark functions listed in Table 3 are used to execute the GST-HBA, HBA, DE, JAYA, and SCA tests. Each algorithm is executed 30 times individually, with 30 being the algorithm dimension and 30 being the population, with a total of 500 iterations. The average convergence curve of various algorithms is demonstrated in Fig. 6. Fig. 6 demonstrates that the convergence curve of the GST-HBA for functions from F1 to F5 and F7 to F12 are lower than other algorithms, demonstrating that it is superior in relation to both fast convergence and optimization precision. When compared to the DE in F6 and F12, DE performs better, but when compared to the conventional HBA, there are clear improvements. The GST-HBA was able to converge to the ideal value even though the convergence accuracy of each technique is not noticeably different from other algorithms for functions F14–F20. In comparison to the original algorithm, GST-HBA has increased both the speed and accuracy of the conventional HBA.

## 7 Antenna S-parameter problem

The result of the antenna S-parameter problem is presented in Table 6.

On seven scalable antenna test functions and one non-scalable antenna test function, this study investigated five optimization algorithms, namely HBA, DE, JAYA, SCA, and our proposed GST-HBA. The scalable function dimension was set to eight, as recommended by Zhang [50], and the non-scalable function's dimension was set to two. A maximum of 500 iterations are used by each algorithm, with a population size of 30. Table 6 reports the outcomes of the function values throughout 30 executions with the best AVG and STD value obtained for each function is highlighted in boldface.

**Fig. 7:**
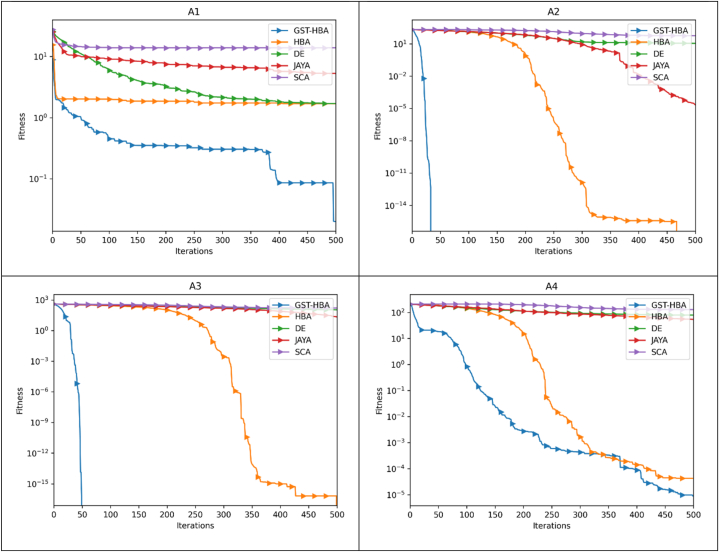

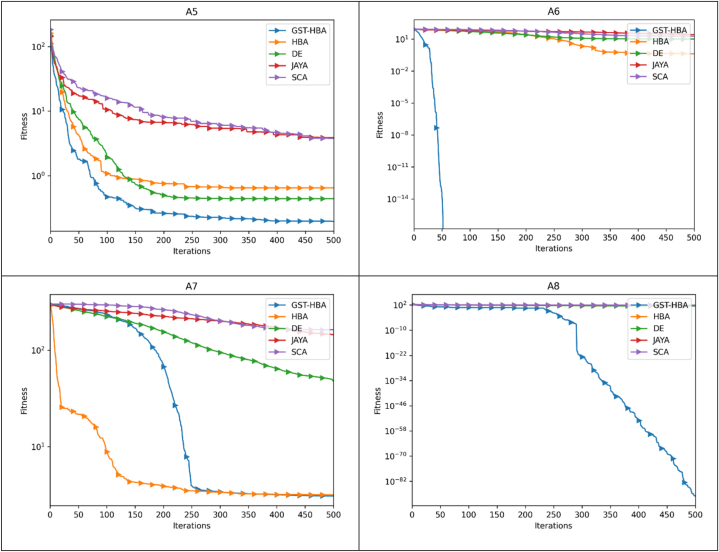
Antenna test suite convergence curve.

In Fig. 7, GST-HBA proves to be a proficient technique in tackling “single-antenna” optimization problems. It exhibits rapid convergence on both A1 and A3 test functions and attains superior objective function values compared to other heuristic methods, namely HBA, DE, JAYA, and SCA. Based on our observations, GST-HBA appears to perform exceptionally well in handling A2, A3, A4, and A5, which are commonly encountered in multi-antenna systems. Notably, it excels in locating the global minimum even when dealing with A5, a challenge that the compared methods seem to find difficult. These findings imply that GST-HBA is an ideal candidate for solving the multi-antenna problem, given the presence of multi-antenna characteristics in A2, A3, A4, and A5. When it comes to tackling A6, A7, and A8 with the isolation characteristic of multi-antenna systems, the effectiveness of GST-HBA is remarkable in terms of its ability to approximate complex landscapes. Notably, its curve exhibits a steady and continuous decrease, which is indicative of its robustness and reliability. Based on Friedman's statistical ranking in Table 6, GST-HBA ranked first and the Wilcoxon test in Table 7 shows the GST-HBA has significant improvement considering the P-value. For the proposed GST-HBA and the compared algorithms, we recorded in Table 5 the number of times each algorithm has the best optimization value compared to others which are denoted by “+”, the number of times each algorithm did not obtain the best optimization value compared to other algorithm denoted by “-” and the number of times each algorithm as a similar result as the compared algorithms are denoted by “ = ”.

**Table 7:** Wilcoxon test for antenna test suite.

GST-HBA vs	–	+	=	R-	R+	P-value
HBA	0	6	2	0	21	2.77E-02
DE	0	8	0	0	36	1.17E-02
JAYA	0	8	0	0	36	1.17E-02
SCA	0	8	0	0	36	1.17E-02

## 8 Uncertainty and limitations associated with antenna S-parameter optimization

Having tested GST-HBA and other state-of-art algorithms on S-parameter benchmark functions that mathematically model the characteristics of different antenna problems with GST-HBA showing promising results, there are still some uncertainties and limitations to consider. While benchmark functions offer valuable insights into the behavior of optimization algorithms in optimizing S-parameters of antennas and finding the optimal S-parameter value for these antennas, they might not fully capture the complexity of real-world antennas. Practical antenna design involves various constraints such as size, weight, cost, and manufacturability, which are not explicitly considered in benchmark functions. Furthermore, the accuracy and validity of benchmark functions in representing actual antenna behavior can vary, requiring careful validation against empirical measurements or simulations. Optimization algorithms can be sensitive to initialization, making it crucial to select appropriate starting points and explore wide parameter space. The computational complexity of the optimization process and potential overfitting to benchmark functions also need to be addressed. Additionally, uncertainties and limitations in the accuracy of the S-parameter model used for optimization can impact the reliability of the optimized antenna design.

## 9 Conclusion

The Golden Sine (GS) Mechanism-based Honey Badger Algorithm (HBA) with Tent Chaos (TC) is a new optimization algorithm introduced in this research (GST-HBA). This algorithm's main goal is to balance exploration and exploitation more effectively during optimization, resulting in rapid convergence and population variety. We ran two different sets of tests including antenna S-parameter optimization to gauge the efficacy of the suggested method. In order to prove our algorithm's supremacy, we also evaluated its performance against that of other optimization algorithms. In the future, this study may investigate the use of the suggested algorithm in other optimization issues, such as feature selection, as well as the hybridization of the algorithm with other approaches to enhance its performance. The limitations of GST-HBA can be categorized into two aspects. Firstly, the complexity of the problem being tackled by GST-HBA plays a significant role. Although the proposed approach has shown promising results in terms of convergence accuracy, improved exploitation, and exploration, it is crucial to acknowledge the influence of problem complexity. Certain problem instances or variations may present challenges that the GST-HBA might struggle to overcome, resulting in suboptimal outcomes. Secondly, the comparative performance of GST-HBA can vary when compared to different optimization algorithms or when applied to real-world scenarios. It is important to consider the effectiveness of the proposed approach in relation to other optimization algorithms and its applicability to diverse real-world scenarios. To address these challenges, future work can explore alternative techniques to further enhance the performance of GST-HBA. For instance, an adaptive parameter strategy can be devised to improve the optimization effectiveness of GST-HBA. Additionally, the evaluation of GST-HBA can be extended to encompass difficult benchmark functions such as multi-objective problems, constrained optimization problems, and image segmentation problems. Additionally, to mitigate these challenges uncertainties, and limitations expressed in section 8, further studies can be made to complement the optimization process with rigorous testing using fabricated prototypes of the optimized antennas, validation against real-world scenarios, and consideration of practical constraints, thereby enabling more reliable and effective antenna designs. Finally, the GST-HBA approach is aimed at improving the realization of more reliable and effective antenna designs, aligning with the demands of contemporary multi-objective, nonlinear, and emerging technological contexts such as Fifth-Generation (5G) systems and the Internet of Things (IoT), we recommend GST-HBA as a tool in the design, simulation, and fabrication to for researchers and practitioners in this field.

## Additional information

No additional information is available for this paper.

## CRediT authorship contribution statement

**Oluwatayomi Rereloluwa Adegboye:** Conceptualization, Formal analysis, Investigation, Methodology. **Afi Kekeli Feda:** Investigation, Methodology. **Meshack Magaji Ishaya:** Methodology, Project administration. **Ephraim Bonah Agyekum:** Methodology, Resources. **Ki-Chai Kim:** Resources, Writing – original draft. **Wulfran Fendzi Mbasso:** Writing – review & editing. **Salah Kamel:** Methodology, Writing – review & editing.

## Declaration of competing interest

The authors declare that they have no known competing financial interests or personal relationships that could have appeared to influence the work reported in this paper.
